# Supplementary material for: Patients’ perceptions of care in a Type 1 hybrid effectiveness-implementation trial on intravenous iron for anaemia in pregnancy in Nigeria
Source: PLoS One. 2026 Feb 2;21(2):e0324578. doi: 10.1371/journal.pone.0324578 (PMC12863501; doi:10.1371/journal.pone.0324578)
Supplement: Attachment — Submitted filename: Response to Reviewers comments_PLOS One.docx [file pone.0324578.s003.docx]

**Point-by-point reviewers' comments and authors' answers**

Title of the manuscript: **Patients’ perceptions of care in a Type 1 hybrid effectiveness-implementation trial on intravenous iron for anaemia in pregnancy in Nigeria**’

**Response to reviewer's comments:**

| **No** | **Reviewers' comments** | **Authors' response** |
| --- | --- | --- |
|  | **Reviewer 1** |  |
| 1. | The title is misleading in that it does not indicate what is actually assessed. The current title: Patient outcomes in a Type 1 hybrid implementation research on intravenous iron for anaemia in pregnancy in Nigeria: a mixed methods study - does not indicate that this is an assessment of perception of the treatment. It rather indicates that it is on treatment outcomes, which it is clearly not. | Thank you for your comments and generally for your review which has helped to improve our paper. Patient outcomes refer to measures of wellbeing that encompass mental health, quality of life, perception of and satisfaction with care, and physical health, as well as the use of services and treatments. However, we have taken on board your title suggestion and that of reviewer 2 and rephrased the title as follows: Patients’ perceptions of care in a Type 1 hybrid effectiveness-implementation trial on intravenous iron for anaemia in pregnancy in Nigeria. |
| 2. | The selection process for participants is not explained - how were participants chosen? It is not entirely clear how many participants were selected and how many dropped out - a flowchart would have been appropriate. | Thank you for your comments. The selection process was explained in the manuscript precisely under the data collection methods for the quantitative aspect of the study, as “Of the 264 participants contacted across all the healthcare facilities, 260 were reachable, and 252 agreed to participate in the exit surveys.” (page 7, lines 172 – 174). For the qualitative aspect, it was stated as “In total, we interviewed 66 participants, purposively selected from all eleven healthcare facilities concurrently with the exit surveys.” (page 8, lines 183 – 184).  For clarity and understanding for the readers, we have included a flowchart as a figure, as suggested, which is referred to as Figure 2 in the manuscript. |
| 3. | The interpretation of the positive perception is likely a reflection of how the protocol was implemented in this study, but it is not clear how well this would be done outside of a study setting. It would have been good to ask if they would have paid for the treatment since cost seems to be an important factor for this delivery method. | Thank you for your comment. For the survey results, we have now rephrased the write-up to replace “perception of treatment” with “perception of care” , which is influenced by both the technical quality of care and the interpersonal aspects of the patient experience, and is more reflective of what we assessed. We also appreciate that the positive perception could have been influenced by the controlled trial setting and may not be reflective of real-world situation and we had noted this as a study limitation. We also clarified in our discussion that participants’ satisfaction likely reflects trial implementation quality (effective counselling, close monitoring, free treatment) rather than intrinsic superiority of IV iron.  We did not ask about willingness to pay as that was outside the scope of our study. We noted as part of our recommendations in the conclusion that “Future implementation studies should evaluate these client outcomes under real-world programmatic conditions, incorporating cost-effectiveness and equity considerations to guide national policy on anaemia management in pregnancy.” |
| 4. | The qualitative results seem quite selective - it may be good to analyze those some more and group the answers on common themes. | Thank you for your suggestion. We went over the transcripts again and arrived at the same themes. This is because we used a deductive approach for our coding based on our indepth interview guide. The guide directly addresses our research questions relating experience and satisfaction with IV iron. |
|  | **Reviewer 2** |  |
|  | **General comment:** I would suggest to the authors that the qualitative results could be presented without drawing broad conclusions from the analysis. Some of the conclusions would require presentation of implementation and service outcomes too and triangulating those with client outcome. I presume will be published in other papers – that said, I think that triangulation of service, implementation and client outcomes together would be a more valuable paper, as I note in general comments below. I have outlined general issues below, followed by specific comments on the various sections | Thank you for your review that has improved our paper considerably. As you already imagined, the service and implementation outcomes of this work have already been published. However, we have triangulated the key findings from the published sub studies with this one in our discussion. We have rewritten the discussion section and removed instances where we have drawn broad conclusions from our qualitative results. |
| 1. | Language: I found the text to be unnecessarily complicated in its explanation. For example, in line 100, “This mixed-methods study employs a convergent parallel design, combining quantitative and qualitative data collection methods to provide a comprehensive perspective on the users' perceptions, experience and satisfaction with IV iron therapy” – an example of language that could be simplified. | Thank you for your comment. We have rephrased this sentence as suggested: “This mixed-methods study combines quantitative and qualitative data collection methods to provide a comprehensive perspective on the users' perceptions, experience and satisfaction with IV iron therapy.” (page 4, lines 102-104). We have also simplified language in other areas of the manuscript. |
| 2. | Two of the limitations noted in the paper – controlled trial setting and recall bias - also serve to highlight that the findings specifically mentioned in this paper are: (i) not representative of the population or the real world setting.; (ii) provide a positive impression of an intervention that has occurred in the past (FCM) against a intervention that is ongoing (oral iron; presumably ongoing, since the women are still pregnant). I’d suggest explicitly considering that when weighing the differences between the interventions. | Thank you for these points. We have rewritten the discussion and clarified that participants’ satisfaction likely reflects trial implementation quality (effective counselling, close monitoring, free treatment) rather than intrinsic superiority of IV iron.  We agree that there are clear differences between the two interventions. FCM given once versus months of treatment for oral iron (this information is now included in the paper in lines 188-190) are intrinsic features of treatment that are non-modifiable and could influence perception of end-users. The single dose was perceived to be advantageous in the qualitative study and we have noted this in the discussion. |
| 3. | Introduction could use more information on the rationale behind the switch from oral to IV iron (noted in line 65-66) and how that informs the design of the tools in this current study. | Thank you for your comment. Lines 98 – 108 detail the rationale behind the switch from oral to IV iron while the justification for our study is presented in lines 124-134. |
| 4. | In my reading, I felt that the authors left many issues unexplored in the quant and qual analysis, which focused only on numbers and percentages – some of the deeper analysis could include association with background characteristics, facilities where they were seen and why, tertiary versus secondary versus primary health care facility. There is an opportunity to go deeper into their health care seeking beliefs and behaviors, and why the mothers feel that way about IV iron in that context, but the analysis remains superficial | Thank you for your suggestion. We have now done further analysis including association of sociodemographic variables with overall perception, which is presented in Table 3. For the qualitative data, we went over the transcripts again and arrived at the same themes. This is because we used a deductive approach for our coding based on our indepth interview guide. The guide directly addresses our research questions relating experience and satisfaction with IV iron. |
| 5. | Rather than a vote counting of how many mothers in each group rated which service, I would suggest a deeper exploration of why the pregnant women said what they said. However, the themes on IV iron – perception, experience , comparison with oral and satisfaction – are superficial and reflect the rationale of why we are using IV as an intervention, i.e. as a response to the challenges that mothers have faced with oral iron and its use. Restating those reasons in the context of a study is always useful, but this is neither new nor innovative and does not move the needle on adoption of IV iron. The practice of presentation of numbers and “vote counting” in the context of the paper is not very useful and subject to bias, as the sampling was purposive. The differences are not meaningful as they could be due to differences in the mothers who were selected into each group using pre-identified criteria | Thank you for your valid comments. We would like to clarify that the participants for the exit surveys were not selected purposively but through systematic random sampling. We used a sampling interval of 4 to select 25% of women and there was no predetermined criteria for their selection. We have made this clearer in the paper: “We aimed to survey 25% of women from each study arm, using systematic random sampling programmed into the data collection platform, to select every fourth participant, resulting in a total of 132 participants in each group and 264 in total”. (page 6, lines 216 – 218).  It was the IDI participants that were purposively selected based on certain characteristics, as described in the manuscript (Page 6, lines 221-226).  The sampling procedure has been further illustrated in Figure 2 as suggested. |
| 6. | As stated, the results gives the reader the impression that one intervention is better than the other, while in fact, it is documenting the perception of pregnant mother vis-a-vis her interaction with the health provider. As it stands, FCM is easy because its ease of administration is a feature, explicitly designed to counter the oft-repeated complaint of the challenges of taking pills throughout pregnancy. I interpret that as a confirmation of the design of the intervention and not reflective of the differences between the interventions | Thank you for catching this. We have rephrased the overall satisfaction written in lines 304-305 to relate to “satisfaction with information and support received from providers” as that was how the question was phrased.  We believe that clients’ perception of the design of an intervention is also relevant. We did not want to assume, for example, that FCM is easy to administer for all women. Some participants in the qualitative study reported dislike for skin piercing, pain and injection phobia.  To address your concern, we have rewritten the discussion section and clarified that participants’ satisfaction likely reflects trial implementation quality rather than intrinsic superiority of IV iron. |
| 7. | Incomplete picture of Type 1 hybrid implementation research: The full picture of implementation should include all outcomes – service, implementation, and client. As a stand-alone paper, while these findings are useful to document, they are neither comprehensive nor insightful. They would be valuable when correlated and presented together with implementation and service outcomes | Thank you for your valid points. Since the service and implementation outcomes of this work have already been published, we have now correlated the key findings from the published sub studies with this one and presented them together in an implementation research framework (figure 3) in our discussion. |
| 8. | Title: I found the title promising but felt that the paper content did not match its promise, as per my comments above. The research framework only features in the introduction, and mixed methods design has multiple issues in interpretation. I’d suggest a simpler descriptive title. | Thank you for your comment. We have taken on board your title suggestion and that of reviewer 1 and rephrased the title as follows: Patients’ perceptions of care in a Type 1 hybrid effectiveness-implementation trial on intravenous iron for anaemia in pregnancy in Nigeria. |
|  | **Specific comments** |  |
|  | **Abstract** |  |
| 1. | Results section – suggest rewriting this section to include more qualitative findings; the quantitative findings are more descriptive than informative | Thank you for your comments. We have rewritten the results section to include more qualitative findings. |
| 2. | Line 44 – it is not the treatment that is rated as excellent (as per table 2); it is the information and services provided around the treatment. Since this is a trial, this speaks more to the deficiencies in the oral iron arm rather than the benefits of the IV iron arm. | Thank you for catching this. This has been deleted here and replaced with result on overall perception of care and it has rephrased in the main paper. |
| 3. | Line 49: I would not state it confidently that this study has established IV iron as a “viable alternative to oral iron”. Consider being more circumspect. | Thank you for this valid comment. This has now been rephrased in the conclusion |
|  | **Introduction** |  |
| 1. | Line 56 – reconsider using AIP as an acronym; it is not a long phrase and AIP is not a standard acronym | Thank you for your suggestion. Anaemia in pregnancy has been written in full throughout the paper. |
|  | **Methods** |  |
| 1. | Line 100-105: See general comments. | Thank you. We have simplified the language in this section. |
| 2. | Line 138 – Please clarify how these participants were selected; “equates to” is not the same as "we selected every fourth participant. | Thank you for the observation. We have made the statement clearer as suggested: “We aimed to survey 25% of women from each study arm, using systematic random sampling to select every fourth participant, resulting in a total of 132 participants in each group and 264 in total”. (page 6, lines 216 – 218) |
| 3. | Line 154 – is this the same as the exit survey? Its confusing -I read this to be another survey; please clarify at the beginning of the methods early on that the data sources are a 10 question exit survey and in-depth interviews | Yes, it is the same survey that was referred to. For clarity, we have made the statement clearer: “For the exit survey, the research team designed a 10-item questionnaire to collect data on participant’s perceptions of care and satisfaction regarding the treatment (FCM or FS) they received during the trial.” (page 7, lines 241 – 243)  For the IDI, we have also rephrased as “Qualitative data were collected through in-depth interviews (IDIs) with the purposively selected participants. A semi-structured topic guide with five sections was developed to explore users' knowledge of anaemia in pregnancy, their perceptions of and relative advantages of IV iron compared to oral iron, and their challenges and experiences with the administration process, including overall satisfaction with IV iron therapy.” (page 7, lines 252 – 255) |
| 4. | Line 161 – Who reviewed it? | The research team reviewed the survey. We have made the statement clearer as “Before data collection, the survey underwent a rigorous review process by the research team, was corrected and was piloted with non-participating pregnant women to ensure the validity and reliability of the tool.” (page 7, lines 248 – 250) |
| 5. | Line 162 - validity and reliability of what exactly? | We were referring to the content of the exit survey in the statement. However, we have made it clearer as “Before data collection, the survey underwent a rigorous review process by the research team, was corrected and was piloted with non-participating pregnant women to ensure the validity and reliability of the tool.” (page 7, lines 248 – 250) |
| 6. | Line 163-164: unclear what the five sections explored? Same as the exit survey or different? | As stated in the manuscript, A semi-structured topic guide with five sections was developed to explore users' knowledge of anaemia in pregnancy, their perceptions of and relative advantages of IV iron compared to oral iron, and their challenges and experiences with the administration process, including overall satisfaction with IV iron therapy.” (page 7, lines 252-255)  The five sections explored:   i. The users’ knowledge about anaemia in pregnancy ii. Their perception of and relative advantages of IV iron compared to oral iron iii. Their challenges with the administration iv. Their experiences of the administration process v. Their overall satisfaction with IV iron as an intervention.   The areas the exit survey assessed were also stated in the manuscript: “The survey assessed trial participants’ perceptions about the communication from providers about their treatment; the challenges experienced during the treatment, including side effects; overall satisfaction with their treatment; and recommendation of treatment to a family member.” (page 7, lines 243 – 246)  The sections that the exit surveyed are:   i. Perceptions of the participants on the communication received from the providers about their treatment ii. The challenges experienced during the treatment, including side effects iii. Their overall satisfaction with the treatment received iv. The recommendation of treatment to a family member |
| 7. | Line 169- All participants or the one in four of the participants who were selected purposively? | Thank you for your question. The participants for the exit surveys were not selected purposively but through systematic random sampling. We used a sampling interval of 4 to select 25% of women and there was no predetermined criteria for their selection. We have made this clearer in the paper: “We aimed to survey 25% of women from each study arm, using systematic random sampling programmed into the data collection platform, to select every fourth participant, resulting in a total of 132 participants in each group and 264 in total”. (page 6, lines 216 – 218).  Please note that it was the IDI participants that were selected purposively based on certain characteristics, as described in the manuscript (Page 6, lines 221-226).  To provide clarify to the text referred to in your question, we have rephrased the sentence to read: “All sampled participants were contacted before their six-week postpartum visit..” (Page 7, line 261) |
| 8. | Line 193 - why was this done? does not add to the value of the data as these participants were purposively sampled | As explained above, the survey participants were selected randomly and not purposively. |
| 9. | Line 208 - given the purposive sampling, i am not sure how these two are complementary, apart from the quantitative findings being a vote count of qualitative findings? These were also done at two different times, as noted in the limitations; would recall bias be an issue For these two methods to be truly complementary, they should be measuring the same underlying construct – for that I would like to see more detailed analysis of the qualitative findings. | Thank you for your observation, which has highlighted the need for clearer communication in our manuscript. We have already clarified that the survey participants were not selected purposively. While the exit survey included both IV and oral iron recipients, the IDI specifically involved women who received IV iron. This deliberate choice aimed to gain a deeper understanding of women’s experiences with IV iron, a treatment approach with its own unique factors, such as administration at healthcare facilities, the infusion process, and associated time commitments. We acknowledge that the differences in two methods, as mentioned in the manuscript; however, we have revised the wording in that section to enhance clarity for our readers.“ In this study, we integrated quantitative and qualitative methods of data collection, focusing on participants who received IV iron, a new intervention with a distinctly different mode of administration compared to oral iron. Here, we considered both data collection methods complementary and of equal significance in our research design.” (page 9, lines 353 – 356) |
| 10. | Line 210 – given previous comments on the authors’ use of quantitative data and the superficial qualitative analysis, the presentation of the joint matrix is not supportive of the convergence of the themes as the resulting convergence could be due to bias in the quantitative results that reflects a superficial qualitative finding. | We appreciate this observation. We have revised the sentence for clarity regarding the specific subset of dataset used for integration “To effectively integrate findings, we employed a joint display matrix which enabled us to systematically compare and contrast survey versus interview findings, highlighting the relationships between the numerical data and the contextual information derived from qualitative data.” (page 9, lines 356 – 417) |
|  | **Results** |  |
| 1. | Line 232-247 and table 2- these findings speak to the implementation of the trial process, rather than the intervention. The comparative interventions are not equivalent for this quantitative analysis. The bivariate tests are really testing the implementation differences rather than intervention differences (and the former is a reflection of the latter – we know that IV and oral iron are different in implementation and we hope that IV iron can address challenges in oral iron). | We appreciate your concerns. We have now rephrased the write-up to replace “perception of treatment” with “perception of care” , which is influenced by both the technical quality of care and the interpersonal aspects of the patient experience. We found as well in the qualitative results under Theme 2: Experience with IV iron administration that “Most women reported having a positive experience with IV iron administration, highlighting the quality of care received from providers.” We now clarify in the discussion that participants’ satisfaction likely reflects trial implementation quality (effective counselling, close monitoring, free treatment) rather than intrinsic superiority of IV iron. |
| 2. | Line 241-242 – the finding of an “easy” treatment could be due to recall bias for a treatment in the past (FCM) as compared to an ongoing treatment (oral iron). | Thank you for your comment. We expanded the qualitative interpretation in the discussion to highlight how participants’ satisfaction reflected both the intrinsic convenience of IV iron and contextual trial factors such as provider communication, education, and follow-up. We noted in the limitations: “Limitations include possible recall bias, particularly for FCM, as participants were interviewed several weeks after treatment (Page 22, lines 615-617) |
| 3. | Line 253 – where were the themes drawn? Only IDI or did some of the exit interview results fed into it? | Thank you for the question. The themes were drawn from the IDI – the procedure is described under the section qualitative data analysis. We have now included that the themes were identified from qualitative analysis. The integration was done subsequently with the survey results as presented in Table 6. |
| 4. | Line 257: did you explore if these positive feelings were engendered as a result of extensive preparation of a pregnant mother for a new procedure like IV iron? | Thank you for your question. We have now rephrased that section to emphasize the reasons for the positive perception of IV iron. |
| 5. | Line 277-286: As noted in comments above, I have reservations of comparing IV and oral iron implementation in parallel. | We appreciate your reservations. However, in this instance the respondents that participated in the IDIs were all in the FCM group. In which case, the perception of the relative advantage of a new treatment against an existing alternative is relevant in our opinion. This was why we explored this in qualitative data collection. |
| 6. | Table 5: As noted in the methods, the convergence elides over the fact that a lot of these are trail related, not intervention related | The clarity of the statement in the methods section has significantly improved the understanding of the results. It effectively illustrates how the quantitative and qualitative findings from the subset of participants who received IV iron are integrated in Table 6. The themes and relevant quotes also speak to patient experiences. |
|  | **Discussion** |  |
| 1. | Line 308 – I would like to see more comprehensive qualitative findings | We appreciate this suggestion. We expanded the qualitative interpretation in the discussion to highlight how participants’ satisfaction reflected both the intrinsic convenience of IV iron and contextual trial factors such as provider communication, education, and follow-up. |
| 2. | Line 311- as noted in the abstract, the stated interpretation of the finding the perception of excellence in the implementation of IV iron with the intervention itself as excellent. | Revised as suggested. We now clarify that participants’ satisfaction likely reflects trial implementation quality (effective counselling, close monitoring, free treatment) rather than intrinsic superiority of IV iron. |
| 3. | Line 317-318 - this theme is a comment on trial implementation, though the finding holds. | Acknowledged. The section was rephrased to clearly state that high satisfaction signifies successful trial implementation and supportive care delivery. |
| 4. | Line 325-327 - The authors’ confidence in this conclusion is not supported by the study design and results. These acceptance rates are artificial, in a trial setting and following on the comments above, “could” is doing the heavy lifting. “Could” is possible if these outcomes, and service and implementation outcomes, are replicated in real life settings. | We have modified and tempered our interpretation and removed conclusive statements. The revised text now emphasizes that these outcomes “indicate” and “suggest” potential for scale-up, contingent upon replication in real-world settings. |
| 5. | Line 337 – inclusion of any quantitative and qualitative findings are necessary but not sufficient for a good mixed methods study. | Thank you for your comment. We have reduced the details on the mixed-methods study in our strengths. |
| 6. | Line 345 – these limitations are critical to why the findings, as currently presented, do not lead to the broader conclusions stated. | Revised Strengths and Limitations section now explicitly links recall bias, controlled setting, and purposive sampling to the cautious interpretation of findings. |
|  | **Conclusion and recommendations** |  |
| 1. | Line 354 – same comment as #4in discussion section | The conclusion now begins with a cautious framing: “adds a critical patient-centred dimension: documenting positive perceptions and high satisfaction,” avoiding “positions as preferred” or “establishes.” |
| 2. | Line 356-358 – same concern for the level of confidence in this conclusion. | The conclusion was rewritten to reflect a measured interpretation, positioning the findings as complementary to other IVON publications and noting that broader adoption depends on addressing contextual barriers. |
