# Supplementary material for: Patients’ perceptions of care in a Type 1 hybrid effectiveness-implementation trial on intravenous iron for anaemia in pregnancy in Nigeria
Source: PLoS One. 2026 Feb 2;21(2):e0324578. doi: 10.1371/journal.pone.0324578 (PMC12863501; doi:10.1371/journal.pone.0324578)
Supplement: Attachment — Submitted filename: Reviewer comment PONE-D-25-22404.docx [file pone.0324578.s002.docx]

**Reviewer comments**

**Manuscript ID PONE-D-25-22404 - Patient outcomes in a Type 1 hybrid implementation research on intravenous iron for anaemia in pregnancy in Nigeria: a mixed methods study.**

General Comments:

The authors of the manuscript titled, “**Patient outcomes in a Type 1 hybrid implementation research on intravenous iron for anaemia in pregnancy in Nigeria: a mixed methods study.**” have conducted a mixed methods study to document pregnant women’s perspectives on IV iron treatment, the study being nested within the IVON trial. While the effort is admirable, I have issues with the central thesis – that the comparison of the two arms (IV and oral iron) in the setting of a trial is informative about the implementation of the IV iron implementation in regular practice. I believe that the design of the study leads to some challenges in interpretation that need to be elucidated in detail, not just mentioned in the limitations section. I read the quantitative results are an indicator of how the trial was implemented, and the qualitative results are a confirmation of why IV iron was developed as an intervention. I would suggest to the authors that the qualitative results could be presented without drawing broad conclusions from the analysis. Some of the conclusions would require presentation of implementation and service outcomes too and triangulating those with client outcome. I presume will be published in other papers – that said, I think that triangulation of service, implementation and client outcomes together would be a more valuable paper, as I note in general comments below. I have outlined general issues below, followed by specific comments on the various sections.

1) Language: I found the text to be unnecessarily complicated in its explanation. For example, in line 100, “This mixed-methods study employs a convergent parallel design, combining quantitative and qualitative data collection methods to provide a comprehensive perspective on the users' perceptions, experience and satisfaction with IV iron therapy” – an example of language that could be simplified.
2) Two of the limitations noted in the paper – controlled trial setting and recall bias - also serve to highlight that the findings specifically mentioned in this paper are: (i) not representative of the population or the real world setting.; (ii) provide a positive impression of an intervention that has occurred in the past (FCM) against a intervention that is ongoing (oral iron; presumably ongoing, since the women are still pregnant). I’d suggest explicitly considering that when weighing the differences between the interventions.
3) Introduction could use more information on the rationale behind the switch from oral to IV iron (noted in line 65-66) and how that informs the design of the tools in this current study.
4) In my reading, I felt that the authors left many issues unexplored in the quant and qual analysis, which focused only on numbers and percentages – some of the deeper analysis could include association with background characteristics, facilities where they were seen and why, tertiary versus secondary versus primary health care facility. There is an opportunity to go deeper into their health care seeking beliefs and behaviors, and why the mothers feel that way about IV iron in that context, but the analysis remains superficial.
5) Rather than a vote counting of how many mothers in each group rated which service, I would suggest a deeper exploration of why the pregnant women said what they said. However, the themes on IV iron – perception, experience , comparison with oral and satisfaction – are superficial and reflect the rationale of why we are using IV as an intervention, i.e. as a response to the challenges that mothers have faced with oral iron and its use. Restating those reasons in the context of a study is always useful, but this is neither new nor innovative and does not move the needle on adoption of IV iron. The practice of presentation of numbers and “vote counting” in the context of the paper is not very useful and subject to bias, as the sampling was purposive. The differences are not meaningful as they could be due to differences in the mothers who were selected into each group using pre-identified criteria.
6) As stated, the results gives the reader the impression that one intervention is better than the other, while in fact, it is documenting the perception of pregnant mother vis-a-vis her interaction with the health provider. As it stands, FCM is easy because its ease of administration is a feature, explicitly designed to counter the oft-repeated complaint of the challenges of taking pills throughout pregnancy. I interpret that as a confirmation of the design of the intervention and not reflective of the differences between the interventions;
7) Incomplete picture of Type 1 hybrid implementation research: The full picture of implementation should include all outcomes – service, implementation, and client. As a stand-alone paper, while these findings are useful to document, they are neither comprehensive nor insightful. They would be valuable when correlated and presented together with implementation and service outcomes.
8) Title: I found the title promising but felt that the paper content did not match its promise, as per my comments above. The research framework only features in the introduction, and mixed methods design has multiple issues in interpretation. I’d suggest a simpler descriptive title.

The comments above are major enough for the authors to consider a revision of how and what they want to present within their manuscript. I have provided specific comments on the various sections in support of my recommendation, with line numbers as indicated.

Specific comments:

Abstract (Pg 3)

1) Results section – suggest rewriting this section to include more qualitative findings; the quantitative findings are more descriptive than informative
2) Line 44 – it is not the treatment that is rated as excellent (as per table 2); it is the information and services provided around the treatment. Since this is a trial, this speaks more to the deficiencies in the oral iron arm rather than the benefits of the IV iron arm.
3) Line 49: I would not state it confidently that this study has established IV iron as a “viable alternative to oral iron”. Consider being more circumspect.

Introduction

1) Line 56 – reconsider using AIP as an acronym; it is not a long phrase and AIP is not a standard acronym

Methods

1) Line 100-105: See general comments.
2) Line 138 – Please clarify how these participants were selected; “equates to” is not the same as "we selected every fourth participant
3) Line 154 – is this the same as the exit survey? Its confusing -I read this to be another survey; please clarify at the beginning of the methods early on that the data sources are a 10 question exit survey and in depth interviews.
4) Line 161 – Who reviewed it?
5) Line 162 - validity and reliability of what exactly?
6) Line 163-164: unclear what the five sections explored? Same as the exit survey or different?
7) Line 169- All participants or the one in four of the participants who were selected purposively?
8) Line 193 - why was this done? does not add to the value of the data as these particiats were purposively sampled
9) Line 208 - given the purposive sampling, i am not sure how these two are complementary, apart from the quantitative findings being a vote count of qualitative findings? These were also done at two different times, as noted in the limitations; would recall bias be an issue For these two methods to be truly complementary, they should be measuring the same underlying construct – for that I would like to see more detailed analysis of the qualitative findings.
10) Line 210 – given previous comments on the authors’ use of quantitative data and the superficial qualitative analysis, the presentation of the joint matrix is not supportive of the convergence of the themes as the resulting convergence could be due to bias in the quantitative results that reflects a superficial qualitative finding.

Results

1) Line 232-247 and table 2- these findings speak to the implementation of the trial process, rather than the intervention. The comparative interventions are not equivalent for this quantitative analysis. The bivariate tests are really testing the implementation differences rather than intervention differences (and the former is a reflection of the latter – we know that IV and oral iron are different in implementation and we hope that IV iron can address challenges in oral iron).
2) Line 241-242 – the finding of an “easy” treatment could be due to recall bias for a treatment in the past (FCM) as compared to an ongoing treatment (oral iron).
3) Line 253 – where were the themes drawn? Only IDI or did some of the exit interview results fed into it?
4) Line 257: did you explore if these positive feelings were engendered as a result of extensive preparation of a pregnant mother for a new procedure like IV iron?
5) Line 277-286: As noted in comments above, I have reservations of comparing IV and oral iron implementation in parallel.
6) Table 5: As note din the methods, the convergence elides over the fact that a lot of these are trail related, not intervention related.

Discussion

1) Line 308 – I would like to see more comprehensive qualitative findings
2) Line 311- as noted in the abstract, the stated interpretation of the finding the perception of excellence in the implementation of IV iron with the intervention itself as excellent.
3) Line 317-318 - this theme is a comment on trial implementation, though the finding holds.
4) Line 325-327 - The authors’ confidence in this conclusion is not supported by the study design and results. These acceptance rates are artificial, in a trial setting and following on the comments above, “could” is doing the heavy lifting. “Could” is possible if these outcomes, and service and implementation outcomes, are replicated in real life settings.
5) Line 337 – inclusion of any quantitative and qualitative findings are necessary but not sufficient for a good mixed methods study.
6) Line 345 – these limitations are critical to why the findings, as currently presented, do not lead to the broader conclusions stated.

Conclusion and recommendations

1) Line 354 – same comment as #4in discussion section
2) Line 356-358 – same concern for the level of confidence in this conclusion.
